# A locus conferring effective late blight resistance in potato cultivar Sárpo Mira maps to chromosome XI

**DOI:** 10.1007/s00122-013-2248-9

**Published:** 2013-12-17

**Authors:** Iga Tomczyńska, Emil Stefańczyk, Marcin Chmielarz, Beata Karasiewicz, Piotr Kamiński, Jonathan D. G. Jones, Alison K. Lees, Jadwiga Śliwka

**Affiliations:** 1 Plant Breeding and Acclimatization Institute-National Research Institute, Młochów Research Centre, Platanowa 19, 05-831 Młochów, Poland; 2 Potato Breeding Zamarte Ltd-IHAR Group, Zamarte 33, 89-430 Kamień Krajeński, Poland; 3 The Sainsbury Laboratory, John Innes Centre, Norwich Research Park, Norwich, NR4 7UH UK; 4 The James Hutton Institute, Invergowrie, Dundee, DD2 5DA Scotland UK

## Abstract

Late blight of potato, caused by *Phytophthora* *infestans*, is one of the most economically important diseases worldwide, resulting in substantial yield losses when not adequately controlled by fungicides. Late blight was a contributory factor in The Great Irish Famine, and breeding for resistance to the disease began soon after. Several disease-resistant cultivars have subsequently been obtained, and amongst them Sárpo Mira is currently one of the most effective. The aim of this work was to extend the knowledge about the genetic basis of the late blight resistance in Sárpo Mira and to identify molecular markers linked to the resistance locus which would be useful for marker-assisted selection. A tetraploid mapping population from a Sárpo Mira × Maris Piper cross was phenotyped for foliar late blight resistance using detached leaflet tests. A locus with strong effect on late blight resistance was mapped at the end of chromosome XI in the vicinity of the *R3* locus. Sárpo Mira’s genetic map of chromosome XI contained 11 markers. Marker 45/XI exhibited the strongest linkage to the resistance locus and accounted for between 55.8 and 67.9 % of variance in the mean resistance scores noted in the detached leaflet assays. This marker was used in molecular marker-facilitated gene pyramiding. Ten breeding lines containing a late blight resistance locus from cultivar Sárpo Mira and the *Rpi*-*phu1* gene originating from the late blight resistant accession of *Solanum* *phureja* were obtained. These lines have extended the spectrum of late blight resistance compared with Sárpo Mira and it is expected that resistance in plants containing this gene pyramid will have enhanced durability.

## Introduction

Late blight, caused by the oomycete pathogen *Phytophthora* *infestans*, is the most economically important disease in potato production worldwide and can only be controlled by frequent application of fungicides (Haverkort et al. 2009). In organic farming systems only fungicides based on copper are permitted. However, from the 1st of January 2006 the maximum allowable application rate in The European Union was restricted to 6 kg of copper per hectare per year (Tresnik 2007) and in Germany this was reduced to 3 kg/ha (Tschöpe et al. 2010).Cultivar resistance to *P*. *infestans* as a strategy for late blight control is gaining its importance (Świeżyński and Zimnoch-Guzowska 2001) due to several factors, including the growing need to produce organic potato crops without the use of copper (Tresnik 2007) and changes to legislation concerning the application of pesticides (Twining et al. 2009).

On average, the traditional potato breeding process takes approximately 12 years from the initial crossing to obtaining a new cultivar due to a laborious selection process including many agronomic and quality traits (Bradshaw 2009). Late blight resistance may be amongst these traits, although it is rarely a priority. Whilst desirable traits like texture, tuber shape and starch content may vary over time according to consumer and industry preferences, the need to obtain potato cultivars with late blight resistance remains constant. Due to changes in virulence of *P*. *infestans* populations, and the corresponding breakdown of host resistance, breeders must continually introduce new sources of resistance.

Sárpo Mira is one of the most late blight resistant table potato cultivars currently available (Kim et al. 2011; White and Shaw 2009). It was developed in Hungary by the Sárvari family and trialed in the UK. Laboratory tests and field trials have demonstrated that Sárpo Mira performs well under conditions of high late blight pressure (White and Shaw 2009; White and Shaw 2010). In 2002, Sárpo Mira was added to the National List in the UK (website: Food and Environment Research Agency http://www.fera.defra.gov.uk). Due to vigorous weed suppressing foliage, a long natural dormancy and the ability to yield well in unfavorable growing conditions, Sárpo Mira has become very popular in organic agriculture and home and allotment gardens in the UK (White and Shaw 2009).

Ten years after official registration, Sárpo Mira’s foliar late blight resistance is still effective, even following the appearance of the *13_A2* *P*. *infestans* genotype (White and Shaw 2009). This highly aggressive genotype was first detected in mainland Europe in 2004 and over subsequent years its frequency increased rapidly, leading to the domination of the British and some European *P*. *infestans* populations by *13_A2* (Cooke et al. 2012). The presence of *13_A2* has been linked with the breakdown of foliar late blight resistance in several cultivars, for example, Lady Balfour, Orla, Setanta and Stirling (Lees et al. 2012). The resistance of these cultivars to *P*. *infestans* was previously recorded as 8 on a 1–9 scale of increasing resistance and declined by 4 or more points when plants were tested using genotype *13_A2*. At the same time, Sárpo Mira showed no change in blight resistance when tested with this *P*. *infestans* genotype (White and Shaw 2009; Lees et al. 2012).

Several aspects of Sárpo Mira’s resistance to late blight have been in the focus of research. Effectoromics was the first approach used to study the complex late blight resistance of Sárpo Mira. Based on the co-segregation of hypersensitive response data obtained with use of RXLR effectors and phenotypic resistance data obtained with differential *P*. *infestans* isolates, it was demonstrated that at least five resistance genes are stacked in Sárpo Mira. Four of the genes, *R3a*, *R3b*, *R4* and *Rpi*-*Smira1* confer qualitative resistance and the fifth, *Rpi*-*Smira2*, has only been detected under field conditions (Rietman et al. 2012). Later, the *Rpi*-*Smira2* has been localized in the *R8* locus on potato chromosome IX and in the field experiments *Rpi*-*Smira2* and *R8* have both mediated quantitative resistance with similar levels of delay in the onset of *P*. *infestans* symptoms. Moreover, the *Avr8* has been reported as *AvrSmira2* supporting the likely identity of the *Rpi*-*Smira2* and *R8* genes (Jo 2013).

The genes *R3a*, *R3b* and *R4* were previously identified in *S*. *demissum* and widely used in potato breeding programs (Rietman et al. 2012). The *R3* gene, which maps to the distal end of potato chromosome XI (El-Kharbotly et al. 1994) has been found to be a complex locus of two functionally distinct *R* genes: *R3a* and *R3b* (Huang et al. 2004). The position of *R4* has not been determined (Verzaux 2010) but because of the similarity to a resistance gene from *S*. *edinense* (*Rpi*-*edn3*), it is likely that *R4* is also located on the long arm of chromosome XI in the *N* cluster (Verzaux et al. 2012). The two other genes, *Rpi*-*Smira1* and *Rpi*-*Smira2*, have been newly identified by Rietman et al. (2012) who postulated that the gene *Rpi*-*Smira1* is a qualitative *R* gene and *Rpi*-*Smira2* is a gene conferring quantitative resistance. These authors proposed a position near the *R3* gene cluster on chromosome XI as the location of the *Rpi*-*Smira1* gene.

Sárpo Mira has also served as a model late blight resistant potato in studies of defense mechanisms. Seven transcripts involved in Sárpo Mira’s resistance reaction to *P*. *infestans* at an early stage of infection were described by Orłowska et al. (2012a). The early induced transcript-derived fragments (TDFs) were homologous to pathogenesis-related (PR) genes and transcription factors. Products of these PR genes have been described previously as playing important roles in signaling pathways, or as able to inhibit pathogen growth. Transcription factors with homology to Sárpo Mira’s TDFs have been shown to regulate the expression of PR proteins in different plant pathosystems (Orłowska et al. 2012a).

In other work, it has been suggested that Sárpo Mira plants use several strategies to obtain high levels of resistance (Orłowska et al. 2012b). The mechanisms responsible for Sárpo Mira’s resistance might rely on the synthesis of a physical barrier against pathogen entry. After infection, leaves become thicker and tougher, which suggests increased synthesis of lignin and callose strengthening cell walls. Leaves also change color to a darker green, which may indicate production of phytoalexins, phenolics and glycoalkaloids. These pathogen-induced phenolic compounds, as well as PR proteins secreted after pathogen infection, are known for their antimicrobial activity. Indeed, extracts made from roots as well as extracts from infected Sárpo Mira leaves strongly inhibit the growth of *P*. *infestans* on rye agar plates (Orłowska et al. 2012b).

Although the presence of at least five stacked resistance genes in Sárpo Mira has been described (Rietman et al. 2012), only a hypothetical chromosomal localization was proposed for what the authors referred to as *Rpi*-*Smira1*. The aim of our work was to further characterize the late blight resistance in Sárpo Mira. Furthermore, we wished to identify molecular markers linked to the resistance locus that would be useful for marker-assisted selection. In this paper, the mapping of the Sárpo Mira resistance locus on chromosome XI of the potato genome is presented. A molecular marker linked to this locus was then applied to pyramid it with an *Rpi*-*phu1* gene from *Solanum* *phureja*. The *Rpi*-*phu1* gene provides good late blight resistance not only in potato leaves, but also in tubers (Śliwka et al. 2006) and plants having this gene show a wider spectrum of resistance to *P*. *infestans* isolates than Sárpo Mira plants (IHAR-PIB, unpublished data). Plants containing both sources of resistance, the late blight resistance locus from Sárpo Mira’s chromosome XI, as described in this study and the *Rpi*-*phu1* gene on chromosome IX, are expected to extend the spectrum, and potentially the durability, of late blight resistance.

## Materials and methods

### Plant materials

One hundred and thirty-seven progeny clones from a cross between cultivar Sárpo Mira (SM, late blight resistance score 9 on 1–9 scale, where 9 = the most resistant; according to The European Cultivated Potato Database (TECPD) (http://www.europotato.org) and the late blight susceptible cultivar Maris Piper (MP, resistance score 3; TECPD), were obtained from The James Hutton Institute, Dundee. The cross (05.Z.165) was carried out in 2005 with Sárpo Mira as the female parent. The two parental cultivars and 137 F1 individuals segregating for resistance according to detached leaflet tests were used to identify the chromosomal location of late blight resistance and for the development of linked markers. Two standard cultivars were also included in detached leaflet tests: foliage blight susceptible Tarpan (resistance score 3; TECPD) and resistant Bzura (resistance score 7; TECPD).

### *Phytophthorainfestans* isolates

Isolates MP324, MP618, MP650 and MP1353 from the collection of the Plant Breeding and Acclimatization Institute-National Research Institute, Młochów (see Table 1 for details) were used to assess foliar resistance. In parallel to the assessments of foliar resistance of test plants, the virulence of all isolates was confirmed on Black’s differential set (Black et al. 1953), which was obtained from SASA, Edinburgh, UK.

**Table 1:** Characteristics (origin, mating type, race defined on Black’s differentials) of *P*. *infestans* isolates used in this study

Isolate	Year, place of origin	Mating type	Race^a^	Resistance^b^			
				cv Sárpo Mira	cv Maris Piper	cv Bzura	cv Tarpan
MP324	1997, Koszalin	A1	1.2.3.4.5.6.7.8.10.11	8.6 ± 0.6	3.0 ± 1.9	7.7 ± 0.8	1.3 ± 0.5
MP618	2005, Nowy Sącz	A2	1.2.3.4.(5).6.7.10.11	8.6 ± 0.8	3.4 ± 1.4	5.7 ± 0.5	2.3 ± 1.2
MP650	2005, Nysa	A2	1.2.3.4.5.(6).7.(8).10.11	8.4 ± 1.0	4.0 ± 1.0	6.5 ± 1.2	3.0 ± 0.9
MP1353	2011, Białuty	A2	1.2.3.4.5.6.7.8.10.11	5.8 ± 0.4	n.t.	2.8 ± 1.2	2.3 ± 0.8

*n*.*t.* not tested

^a^Race nomenclature is based on ability to infect the Black’s differential potato set (potatoes carrying *R1*–*R11* genes) (Black et al. 1953)

^b^The mean resistance of cv. Sárpo Mira (resistant parent), Maris Piper (susceptible parent) and two standard cultivars Bzura and Tarpan to each of the isolates is shown in 1–9 scale, where 9 is the most resistant ± standard deviation

For phenotyping of the mapping population isolates MP324, MP618 and MP650 were used. All of the isolates were of complex race. However, in contrast to MP324 and MP650, the isolate MP618 was not able to overcome the resistance conferred by *R8*. Virulence to *R6* was detected in isolates MP324 and MP618 but not in all tests done with isolate MP650. None of these isolates was able to infect *R9*.

For the gene pyramiding study isolates MP324 and MP1353 were used. Although virulence profiles on Black’s differentials of these two isolates were the same, the isolate MP324 was avirulent to Sárpo Mira and MP1353 was able to overcome Sárpo Mira’s resistance (Table 1). None of the isolates was able to defeat the resistance conferred by the *Rpi*-*phu1* gene.

### Late blight resistance assessment

Late blight resistance was evaluated in three subsequent years (2010–2012) in laboratory tests conducted on detached leaflets of the parental clones and the mapping population. In each year, each isolate was tested on three leaflets per plant genotype, in duplicate and on two separate dates. In total, 36 leaflets per genotype and per isolate were assessed.

Plants of progeny and parental clones were grown from tubers and maintained in a glasshouse. Leaflets were collected from the middle part of fully developed leaves from 6-week-old greenhouse-grown plants and placed on wet paper with the abaxial side up (Zarzycka 2001).

Before each resistance test *P*. *infestans* isolates were multiplied on leaves of the susceptible cultivar Tarpan. Sporangia were released from the surface of the leaves by irrigation with sterile distilled water. The resulting sporangial suspensions were examined microscopically and the sporangial concentration adjusted to 5 × 10^4^/ml. Zoospore release was stimulated by incubation of the suspension for 3 h at 5 °C.

A single 30 μl droplet of the inoculum was deposited on the abaxial side of each tested leaflet. After inoculation, leaflets were kept in a climatic chamber and exposed to conditions conducive for disease development (high relative humidity, 16 °C and the first 24 h in darkness and then in constant light of approximately 1.600 lx). After 24 h, the leaves were turned over, the adaxial side up and after 7 days of incubation, resistance was evaluated by scoring leaflets on a 1–9 scale, where 9 is the most resistant (Zarzycka 2001).

### DNA isolation and sequence-specific markers

Genomic DNA was extracted from 1 g of fresh young leaves of each of the 137 progeny and two parent plants grown in the glasshouse, using the DNeasy Plant Maxi kit (Qiagen, Hilden, Germany) according to the manufacturer’s instructions.

We hypothesized that the resistance gene cluster on chromosome XI would be a likely location of Sárpo Mira’s quantitative foliar resistance (Tomczyńska and Śliwka 2010; White and Shaw 2010). This hypothesis was supported by the presumed location of *Rpi*-*smira1* (Rietman et al. 2012). Three sources of primers were applied to the SM × MP mapping population, namely: Sol Genomics Network website (The Tomato-EXPEN 2000 genetic map: http://solgenomics.net), literature and the potato genome sequence from the Potato Genome Sequencing Consortium website (http://www.potatogenome.net) (Table 2).

**Table 2:** Markers and primers used in this study, their sequences, annealing temperatures and restriction enzymes used to detect polymorphism

Name of the marker	Primer sequences	Ta (^o^C)	Product size (bp)	Restriction enzyme	Source
B11.6		60	1,800	*Eco*RI	Polish patent no: P-399117
GP38	TGGAACTTACTTCACTGACAAACT TGCAGTAACTGAAAGCAACAGAT	55	800	*Rsa*I	Marczewski et al. 2006
cLEC-24-C3	AGATCGGCAAATGATCCAAG ACTTGTGGCGAAAAATGAGG	55	1,200	*Taq*I	SGN^c^
C2_At1g56450	ACTTGTTCTTGGTGGAGTAAAAAATGG ACTCCCTCTTCTGTGATTTTTGCAATCTG	55	1,000	*Eco*RI	SGN^c^
C2_At5g60540	TGCTGTTTTCATCCGTGCTCC AGTTAATTCGGGATGAAAAGCAG	55	900	*Msp*I	SGN^c^
C2_At3g52090	AGGGATACGAAGATCATGAATGCAGC ACTCTTCAGATGATCAAGTTCCTTGTC	55	1,500–1,400	a.s.^a^	SGN^c^
C2_At1g07960	ATGGTTTGTCAAATTTTGTGTTCC AAGAGTTTGAATGTAGGGTATGAATG	55	800	*Hinf*I	SGN^c^
C2_At5g60600	TTGCTTCAAGGTTGCAGAATGCG ACCAGGCAAGTGTGACGTCTTCTCTC	55	900	*Hha*I	SGN^c^
cLET5E4	CCAGGCATGCTCAATTTGGAGT TTCCCTGTTTGGACTACTTGTGGA	55	300	*Hha*I	Huang et al. (2005)
45/XI	AGAGAGGTTGTTTCCGATAGACC TCGTTGTAGTTGTCATTCCACAC	55	900–1,500	a.s.^a^	PGSC^d^ chr11:39956309..39958308
123/XI	TCCCATAACGATCTCCCAAA TTTGCTCCTTACCCATCACC	60	1,500	*Taq*I	PGSC^d^ chr11:41151558..41153557
phu6	AGAGACCCTGGATATATTTCATAGCTCT CGCTCTAGGCACAGGGCTCAATGCTGAT	55	298	SCAR^b^	Śliwka et al. (2013)

^a^Allele-specific

^b^SCAR-sequence characterized amplified regions

^c^SGN-www: SOL Genomics Network Database

^d^PGSC-www: Solanaceae Genomics Resource Genome Browser Potato (*Solanum* *phureja* clone DM1-3 516R44) PGSC v2.1.11 Pseudomolecules

DNA of parental cultivars and 137 progeny plants was amplified using the following conditions. The reaction mixture of 20 μl contained 2 μl of 10× Taq PCR buffer Mg^2+^ Plus, the four deoxynucleotides (0.1 mM; Sigma-Aldrich, St. Louis, MO, USA), primers (0.2 μM; Sigma-Aldrich, St. Louis, MO, USA), *Taq* DNA polymerase (0.05 U/μl; GenoPlast Biochemicals) and 10–30 ng of the template DNA of each of 137 progeny and parent plants.

The PCR program for the annealing temperature 60 °C was: 93 °C–180 s; 39 cycles of: 93 °C–45 s, 60 °C– 45 s, 72 °C–90 s; 72 °C–420 s, the PCR program for the annealing temperature 55 °C was: 94 °C–180 s; 39 cycles of: 94 °C–15 s, 55 °C–15 s, 72 °C–60 s; 72 °C–420 s. The annealing temperature for each marker is indicated in Table 2.

Clear single-band PCR products that were identical for SM and MP were digested with a set of 19 frequently cutting restriction enzymes (*Eco*RI, *Msp*I, *Hha*I, *Dra*I, *Xap*I, *Alu*I, *Rsa*I, *Fsp*BI, *Cfr*13I, *Bsh*1236I, *Hinf*I, *Hin*dIII, *Tru*1I, *Tas*I, *Taq*I, *Tai*I, *Hpy*F3I, *Mva*I, *Bsu*RI) to detect polymorphisms. The reactions of PCR products with corresponding restriction endonucleases (Fermentas Life Sciences, Thermo Fischer Scientific Inc.), listed in Table 2, were performed according to the manufacturer’s recommendations.

PCR and digestion products were separated in a 1.5 % agarose gel and visualized after staining with ethidium bromide under UV transillumination.

If a polymorphism was revealed between the parents, the marker was subsequently tested on a set of five resistant and five susceptible individuals of the progeny. Markers found to be linked to resistance were tested on DNA of the entire mapping population.

### Gene pyramiding

Cultivar Sárpo Mira was crossed with two donor clones of the *Rpi*-*phu1* gene: Z-03.3817 and Z-03.3827. From these crosses 83 pedigree lines were obtained in which the presence of resistance genes originating from both parents was examined using markers. The presence of the resistance locus from Sárpo Mira’s chromosome XI was examined using marker 45/XI and the presence of the *Rpi*-*phu1* gene using marker phu6 (Table 2). The PCR program for phu6 primers was: 94 °C–180 s; 39 cycles of: 94 °C–30 s, 55 °C–45 s, 72 °C–45 s; 72 °C–420 s.

Results obtained with molecular markers were then confirmed using detached leaflet tests conducted in 2012 on two dates with two replicates and three leaflets per genotype per replicate. Each test was done with two *P*. *infestans* isolates: MP324 and MP1353 (Table 1).

### Statistical and linkage analyses

The comparison of a frequency distribution of the values in the phenotypic data to a Gaussian normal distribution was checked by the Kolmogorov–Smirnov test. A Chi-square test was applied to compare the obtained and expected segregation ratios.

The correlation between the mean resistance values obtained with the three *P*. *infestans* isolates (MP324, MP618, MP650) in 2010–2012 was evaluated by calculating the linear Pearson’s correlation coefficients.

Marker-trait linkages were estimated by the Student’s *t* test. Determination coefficients (*R* ^2^) were calculated on the basis analysis of variance by comparing sums of squares of the given factor/marker to the total sum of squares.

All statistical analyses were performed using computer program STATISTICA for Windows (Stat Soft, Inc., Tulsa, OK, USA).

A map of a single linkage group of chromosome XI was constructed of markers linked to the resistance and to each other. Linkage analyses were performed using JoinMap^®^ 4 (Van Ooijen 2006) with the following settings: CP population type, independence LOD as a grouping parameter (linkages with LOD > 3 were considered significant), regression mapping algorithm and Haldane’s mapping function.

## Results

### Late blight resistance assessment

Sárpo Mira was resistant to *P*. *infestans* isolates MP324, MP618 and MP650 and susceptible to isolate MP1353, while Maris Piper was susceptible to all tested isolates (Table 1). The mean resistance scores of the two cultivars differed by 4.6–5.6 points on the 1–9 scale depending on the *P*. *infestans* isolate applied. The results of resistance testing of the standard cultivars were as expected: Tarpan was susceptible and Bzura was resistant or moderately resistant in all tests (Table 1).

The mean, 2010–2012, results of detached leaflet resistance tests obtained for the mapping population using three *P*. *infestans* isolates were strongly correlated with each other. Pearson correlation coefficients between mean resistance values were as follows: *r* = 0.97 (MP324/MP618), *r* = 0.87 (MP324/MP650), *r* = 0.89 (MP618/MP650), *p* < 0.05. There was also a strong correlation between the mean detached leaflet resistance results from different years within an individual isolate (data not shown).

Analysis of variance showed that plant genotype had the strongest influence on resistance scores, explaining more than 50 % of the variation observed in resistance tests carried out using isolate MP650 and more than 60 % for isolates MP324 and MP618. However, the effects of the year and genotype × year interaction were also significant (Table 3).

**Table 3:** Analysis of variance in mean resistance scores in the mapping population of SM × MP repeated in two dates in 2010–2012 with three *P*. *infestans* isolates

Factor	*df* ^a^ effect	Mean sum of squares effect	*df* error	Mean sum of squares error	*F*	*p*	*R* ^2 b^
MP324							
{1} year	2	737.70	4,461	1.71	431.17	0.0000	5.00
{2} genotype	136	133.50	4,461	1.71	78.03	0.0000	61.54
Interaction: 1 × 2	267	8.37	4,461	1.71	4.89	0.0000	7.57
MP618							
{1} year	2	698.47	4,461	1.25	557.41	0.0000	5.71
{2} genotype	136	113.09	4,461	1.25	90.25	0.0000	62.94
Interaction: 1 × 2	267	7.75	4,461	1.25	6.18	0.0000	8.47
MP650							
{1} year	2	430.85	4,462	1.24	345.92	0.0000	4.26
{2} genotype	136	75.28	4,462	1.24	60.44	0.0000	50.58
Interaction: 1 × 2	267	13.42	4,462	1.24	10.77	0.0000	17.70

^a^Number of degrees of freedom

^b^Percent of variance explained

**Fig. 1:**
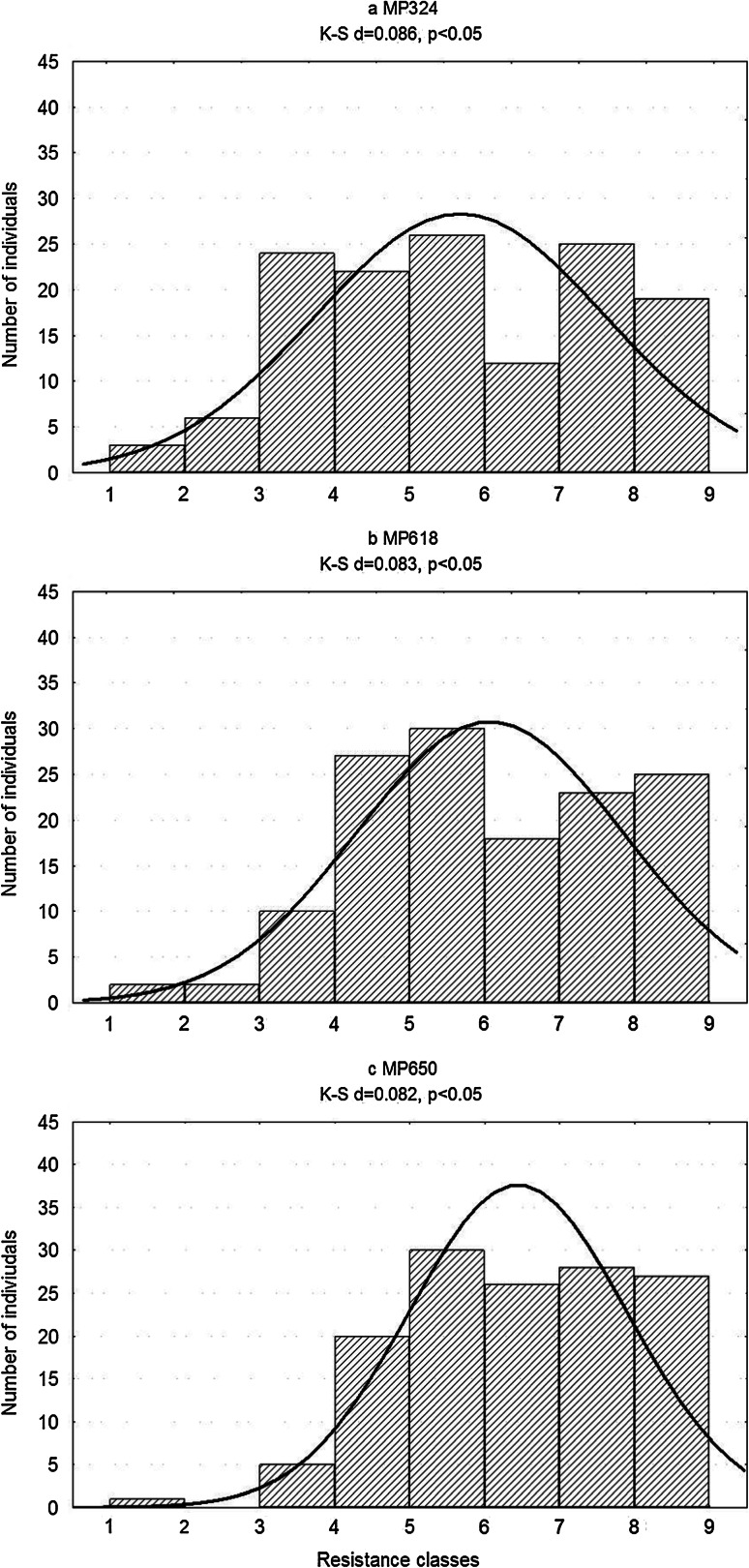
Frequency distribution of mean resistance to *P*. *infestans* (three isolates: a MP324, b MP618, c MP650) of detached leaflets in the population SM × MP in 2010–2012. The resistance was evaluated on 1–9 scale, where 9 was the most resistant. The fitness to the normal curve: *K–S* Kolmogorov–Smirnov test, *d* coefficient calculated for this test, *p* probability, the line indicates the normal curve

Distributions of mean late blight resistance scores of the mapping population deviated significantly from normality, as confirmed by the Kolmogorov–Smirnov test (Fig. 1). The range of late blight resistance scores in SM × MP progeny was between 1.0 and 9.0. The division between resistant and susceptible classes was not clear. For results obtained with isolates MP324 and MP618, as can be seen in Fig. 1a, b, two overlapping groups of resistant and susceptible individuals caused weak bimodal distributions. Moderately susceptible individuals were observed. In the tests conducted with isolate MP650, the results were skewed towards resistance and their range was limited to 3–9 with one very susceptible exception (Fig. 1c).

### Mapping of Sárpo Mira resistance

We found 11 simplex polymorphic markers segregating in a Sárpo Mira × Maris Piper population and linked to resistance against all tested isolates (Table 2). All of the markers segregated in a 1:1 (*χ* ^2^ test, *p* < 0.105 to *p* < 0.667).

**Fig. 2:**
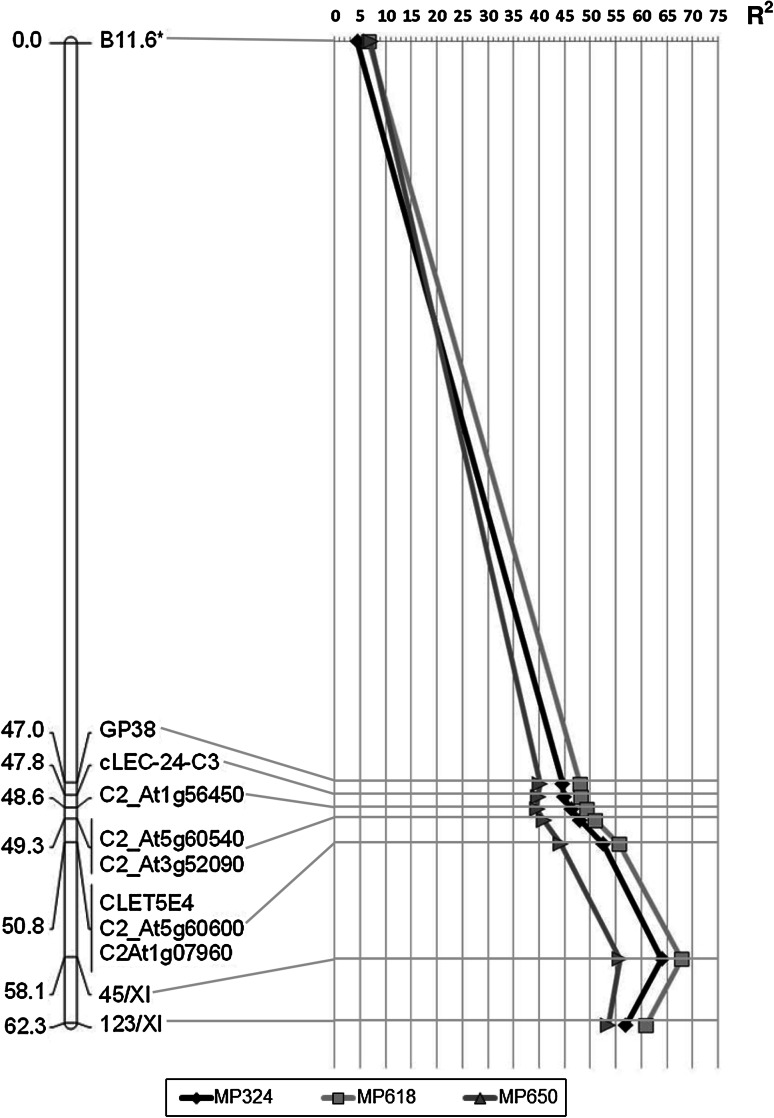
Distribution of percentages of variance (*R* ^2^) in resistance tests with three *P*. *infestans* isolates (MP324, MP618, MP650) explained by markers along Sárpo Mira chromosome XI. All markers are significantly linked to late blight resistance at *p* < 0.0000. The only exception is marked as *. In this case linkage is significant with *p* < 0.01 to *p* < 0.002 depending on *P*. *infestans* isolate used in tests. On the left, genetic distances in cM are given

Using these 11 markers, a genetic map of chromosome XI of Sárpo Mira, where the resistance to these isolates is located, was constructed (Fig. 2). Five of the markers were redundant because no recombinants were present in the population.

Ten of the markers were strongly linked to the resistance scored in our tests (Student’s *t* test, *p* < 0.000). The remaining marker, B11.6, although located at 47 cM distance from the marker GP38 still showed a significant, but weaker, association with the resistance (Student’s *t* test, from *p* < 0.010 to *p* < 0.002 depending on *P*. *infestans* isolate applied for the resistance test).

**Fig. 3:**
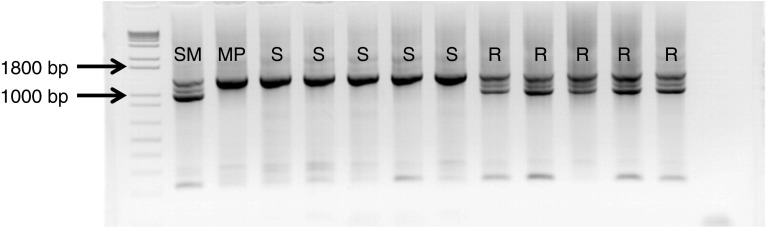
The band pattern of PCR marker, 45/XI, suggested the presence of resistance locus. *SM* Sárpo Mira, resistant parent; *MP* Maris Piper, susceptible parent; *R*, *S* (resistant, susceptible) phenotype of progeny inoculated with three *P*. *infestans* isolates. Sizes of polymorphic bands are indicated on the left

The variance in the resistance test results obtained with all three *P*. *infestans* isolates, explained by marker-trait linkages, is presented in Fig. 2. The largest determination coefficient was reached for the marker 45/XI (Fig. 3): 55.8 % for MP650 results, 67.9 % for MP618 and 64 % for MP324. The markers (C2_At1g07960, C2_At5g60600, cLET5E4, 123/XI) explained 44–57 % of the variance depending on the marker and *P*. *infestans* isolate. This showed that the resistance locus was situated between these flanking markers, somewhere in the vicinity of the region where marker 45/XI is situated.

The marker B11.6, the weakest associated with a trait, explained less than 7.1 % of phenotypic variance. This suggests that this marker is located outside the region which has influence on the late blight resistance.

### Gene pyramiding

**Fig. 4:**
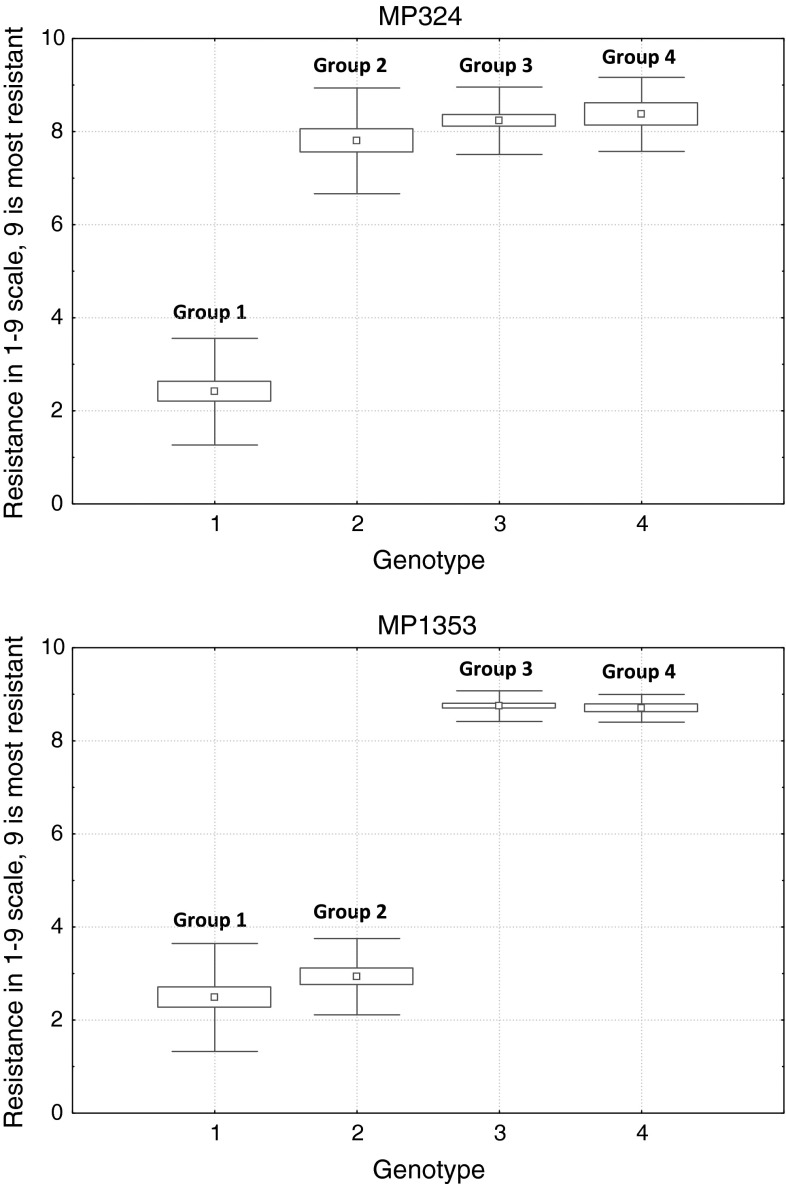
Box plots illustrating resistance profiles of four different groups of progeny Sárpo Mira × clones Z-03.3817 and Z-03.3827. Genotypes were tested with use of two *P*. *infestans* isolates MP324 and MP1353. The *square* indicates the mean value, *box* around it indicates standard error and the *whiskers* indicate standard deviation

The 83 potato lines obtained from crosses between Sárpo Mira and *Rpi*-*phu1* donors were tested for the presence of resistance genes by marker-assisted selection and detached leaflet tests, allowing four groups of genotypes to be classified: group 1 (without any resistance genes, *N* = 26), group 2 (with the locus on Sárpo Mira’s chromosome XI, *N* = 19), group 3 (with the *Rpi*-*phu1* gene, *N* = 28), group 4 (with the locus on Sárpo Mira’s chromosome XI and the *Rpi*-*phu1* gene, *N* = 10). In tests done with both isolates MP324 and MP1353, the lowest mean resistance value was observed in the group of genotypes where none of resistance genes was found (group 1, Fig. 4). Resistance of group 2 genotypes was dependent on the isolate used in the test: only isolate MP1353 was able to overcome the resistance provided by Sárpo Mira’s locus on chromosome XI. Comparing mean resistance scores obtained for group 1 and group 2 with isolate MP1353, higher mean scores for group 2 were observed, however, the difference was not significant. The highest mean resistance scores were observed for group 3 and 4 genotypes in tests with both isolates (Fig. 4). As none of the isolates used in this study was virulent on plants with *Rpi*-*phu1* gene, the presence of both Sárpo Mira chromosome XI resistance locus and *Rpi*-*phu1* gene together was confirmed by molecular markers.

We compared the MAS results with the phenotypic assessment and two recombinants between marker 45/XI and the resistance locus from Sárpo Mira’s chromosome XI (group 2) were detected. In both cases, we detected the presence of the marker allele, indicating the presence of the resistance allele, which was not confirmed by the phenotyping. Contradictory results of resistance tests and phu6 marker (group 3 and 4) were detected in the case of 11 individuals. The majority of these (*N* = 9) were false-positive genotyping results, where the marker was amplified in susceptible individuals. Two cases were the opposite: the phu6 marker was not detected, but the results of detached leaflet assays with both MP324 and MP1353 *P*. *infestans* isolates showed that the plants were resistant and contained the *Rpi*-*phu1* gene.

## Discussion

This study provides direct evidence for the chromosomal position of a locus in cultivar Sárpo Mira conferring foliar late blight resistance to *P*. *infestans* isolates representative of the Polish pathogen population. Distributions of mean late blight resistance scores of the mapping population were weakly bimodal (Fig. 1), suggesting that one locus in simplex form was most effective in our tests. These distributions were blurred by the presence of individuals that exhibited diverse levels of quantitative resistance and only a small number of very susceptible ones. Still, our results support an essential role of a single locus in Sárpo Mira detached leaflet assay resistance, in agreement with data cited by Orłowska et al. (2012b). In trials conducted at the LKF-Vandel Breeding Foundation, populations obtained from crosses with Sárpo Mira as a parent showed a 1:1 segregation of resistant and susceptible individuals, suggesting that most of Sárpo Mira’s field resistance to the current Danish *P*. *infestans* population is also conferred by one *R* gene, or closely linked *R* genes (Orłowska et al. 2012b). In our assays for late blight resistance in the mapping population, three *P*. *infestans* isolates were used (Table 1). Their virulence spectra against Black’s differentials were broad and complementary. The resistance data obtained were strongly correlated, indicating that the same broad spectrum resistance locus is effective against all three isolates of pathogen. Therefore, we aimed to map this crucial locus and find molecular markers that would facilitate its introduction through potato breeding. Such a strategy would ensure the exploitation of the most effective resistance locus of Sárpo Mira. However, the resistance of this cultivar is complex and affected by many genetic factors.

For mapping purposes, we focused on markers linked in coupling with resistance test results because potato is an autotetraploid species with tetrasomic inheritance. This made the search for suitable markers more challenging, since on average only one quarter of the polymorphic and segregating markers was in the desired linkage phase. As proposed earlier, markers from potato chromosome XI were analyzed (Tomczyńska and Śliwka 2010; White and Shaw 2010; Rietman et al. 2012). Indeed, 11 markers linked to the resistance in the SM × MP mapping population were identified and used to generate a 62.3 cM long genetic map of one linkage group of Sárpo Mira’s chromosome XI (Fig. 2). Marker 45/XI explained that most of the variance observed in late blight resistance tests (up to 68 %) and can be used as a good predictor of late blight resistance in selection (Fig. 3). Interestingly, primers for the marker 45/XI were designed on the basis of an *S*. *phureja* sequence annotated as disease resistance protein I2 (PGSC genome browser v2.1.11, chr11:39956309..39958308). The *I2* gene confers resistance to *Fusarium* *oxysporum* f. sp. *lycopersici* in tomato and there is high colinearity between the *R3* region from potato and the *I2* locus from tomato (Huang et al. 2005). It has been evidenced that the R3a protein is more related to the I2 protein than to other known late blight R proteins (Huang et al. 2005). This indicates that the marker 45/XI is located in the vicinity of the *R3* locus.

In the *R3* gene cluster on chromosome XI several late blight resistance genes originating from *S*. *demissum* are located close to each other: *R3* (*R3a* and *R3b*), *R5*, *R6*, *R7*, *R9*, *R10* and *R11* (El-Kharbotly et al. 1994; Huang 2005; Huang et al. 2005; Bradshaw et al. 2006). However, the strong effect on resistance described in our study is most likely not caused by any of the listed genes. It is more likely that a new *R* gene homolog, located in the *R3* gene cluster, which may correspond to *Rpi*-*Smira1* (Rietman et al. 2012), is responsible for the effective resistance observed in our tests. We provide the following arguments in support of this assertion.

All *P*. *infestans* isolates used in our study were able to infect plants with *R3*, *R4*, *R5*, *R7*, *R10* and *R11,* so these genes can be excluded as the ones conferring resistance observed in the SM × MP mapping population. Moreover, a marker derived from the *R3a* gene was tested in a sample of our mapping population and did not show linkage to the resistance results (data not shown).

Isolate MP1353, which was avirulent on *R9*, could infect Sárpo Mira, supporting the hypothesis that *R9* is also not responsible for the resistance of Sárpo Mira in this study. This result corresponds with that of Rietman et al. (2012) who found that the only isolate able to infect potato genotypes having the *R9* gene was PIC99177, to which Sárpo Mira is resistant.

Virulence profiles of isolates used in the study of Rietman et al. (2012) indicated that resistance in Sárpo Mira observed in his tests could potentially be conferred by the *R6* gene. Of nine isolates used in detached leaflet tests, only isolate IPO-C was able to infect Sárpo Mira or plants of Black’s differential set with the *R6* gene (Rietman et al. 2012). However, all isolates used in our tests were able to fully (or partially, in case of MP650) infect plants with the resistance gene *R6* and Sárpo Mira was resistant to these isolates, indicating that its defense did not rely on Avr6 recognition.

Nevertheless, *S*. *demissum* *R* genes *R3* and *R4* present in Sárpo Mira, could be contributing to its quantitative late blight resistance: findings from other studies suggest that *R* genes, even if they have been overcome by a compatible isolate of the pathogen, may perform as a quantitative trait locus (Gebhardt and Valkonen 2001; Stewart et al. 2003; Pilet et al. 2005; Tan et al. 2008; Danan et al. 2009; Rauscher et al. 2010). These defeated R genes are able to delay disease initiation and provide rate-reducing resistance, referred to as a ghost or residual effect. When plants were inoculated with an isolate compatible to defeated genes, a small but significant level of late blight resistance was reported in the case of the *R1*, *R10*, *R11* genes (Stewart et al. 2003), the *R2* gene (Pilet et al. 2005) and a gene originating from *Solanum* *berthaultii* (Rauscher et al. 2010). It was also confirmed that greater field resistance was observed in the case of potato cultivars with *R1* and *R3* genes from *S*. *demissum* than in genotypes lacking such genes (Khavkin et al. 2010).

Although the identity of the gene or genes underlying Sárpo Mira qualitative resistance located on chromosome XI remains unknown, this locus is valuable for potato breeding programs. Gene pyramiding remains a promising approach to obtain durable and broad spectrum resistance. The success of pyramiding three late blight resistance genes (*Rpi*-*sto1*, *Rpi*-*vnt1*.*1* and *Rpi*-*blb3*) was demonstrated by Zhu et al. (2012) who showed that the resistance spectrum of 23 transformed plants was equivalent to the sum of the spectra of the individual *R* genes with no silencing, or any other negative effects, affecting the function of the inserted genes.

We aimed to combine Sárpo Mira’s resistance conferred by the locus on chromosome XI with resistance from *S*. *phureja* to weaken selection pressure on the pathogen population for strains virulent towards each of the resistance sources and to enhance the durability of the overall resistance.

*The* *Rpi*-*phu1* gene from *S*. *phureja* is the major gene located on chromosome IX. Importantly, it is not correlated with a long vegetative period (Śliwka et al. 2006) and it confers broad spectrum resistance: only isolate EC1 from Ecuador has been shown to be able to defeat this gene (Foster et al. 2009). The *Rpi*-*phu1* gene has not yet been released in a potato cultivar and there has, therefore, been little selection pressure on the *P*. *infestans* population towards virulence.

With molecular markers linked to the Sárpo Mira resistance locus on chromosome XI and a marker derived from the resistance gene originating from *S*. *phureja*, we were able to identify with high probability 10 plant genotypes in which both genes were stacked, and to confirm the result using detached leaflet tests. Within 83 progeny lines there were 2 recombinants between marker 45/XI and the locus on chromosome XI from Sárpo Mira in group 2. However, this number may be actually higher due to a masking of the effect of the Sárpo Mira’s locus by the *Rpi*-*phu1* gene in the pyramid. Distinguishing plants from group 4 carrying both genes was possible with the use of markers. There were some false-positive results between phenotypic tests and MAS with the use of the phu6 marker. Although primers for phu6 amplify the sequence inside the resistance gene, this amplified fragment may be related not only to *Rpi*-*phu1* but also with its homologs, which do not confer effective resistance. Because the markers used for selection still produce some false results, more reliable and closely linked (in case of 45/XI) markers are desirable. However, the use of markers in combination with phenotypic disease resistance tests allowed us to make progress in breeding potatoes with the stacked genes from two late blight resistance sources.

With the accumulation of several resistance genes in one plant genotype, expression of these genes is simultaneous and they may mask each other’s effects. The ultimate verification of the marker-assisted selection results would be obtained by infecting the selected plants with a *P*. *infestans* isolate able to overcome the resistance conferred by the *Rpi*-*phu1* gene, for example isolate EC1 as reported by Foster et al. (2009). Despite our best efforts, we have not been able to obtain an isolate of EC1 with satisfactory aggressiveness characteristics with which to carry out a reliable test, or with the ability to consistently overcome *Rpi*-*phu1*.

The biological activity of *R* genes depends significantly on genetic background (Kim et al. 2011), so predicting the phenotypic effect of resistance genes in a pyramid is difficult. The agronomic value of promising individuals carrying the late blight resistance locus from Sárpo Mira’s chromosome XI and the *Rpi*-*phu1* gene will be assessed in field trials. These tests will verify the durability of the resistance over time.

## References

[CR1] Black W, Mastenbroek C, Mills WR, Peterson LC (1953). A proposal for an international nomenclature of races of *Phytophthora**infestans* and of genes controlling immunity in *Solanum**demissum* derivatives. Euphytica.

[CR2] Bradshaw JE (2009). Potato Breeding at the Scottish Plant Breeding Station and the Scottish Crop Research Institute: 1920–2008. Potato Res.

[CR3] Bradshaw JE, Bryan GJ, Lees AK, McLean K, Solomon-Blackburn RM (2006). Mapping the R10 and R11 genes for resistance to late blight (*Phytophthora**infestans*) present in the potato (*Solanum**tuberosum*) R-gene differentials of Black. Theor Appl Genet.

[CR4] Cooke DEL, Cano LM, Raffaele S, Bain RA, Cooke LR (2012). Genome analyses of an aggressive and invasive lineage of the Irish potato famine pathogen. PLoS Pathog.

[CR5] Danan S, Chauvin JE, Caromel B, Moal JD, Pellé R, Lefebvre V (2009). Major-effect QTLs for stem and foliage resistance to late blight in the wild potato relatives *Solanum**sparsipilum* and *S*. *spegazzinii* are mapped to chromosome X. Theor Appl Genet.

[CR6] El-Kharbotly A, Leonards-Schippers C, Huigen DJ, Jacobsen E, Pereira A, Stiekema WJ, Salamini F, Gebhardt C (1994). Segregation analysis and RFLP mapping of the *R1* and *R3* alleles conferring race-specific resistance to *Phytophthora**infestans* in progeny of dihaploid potato parents. Mol Gen Genet.

[CR7] Foster SJ, Park T-H, Pel MA, Brigneti G, Śliwka J, Jagger L, Vossen EAG, Jones JDG (2009). *Rpi*-*vnt1*.*1*, a *Tm*-*2*^*2*^ homolog from *Solanum**venturii* confers resistance to potato late blight. Mol Plant Microbe Interact.

[CR8] Gebhardt C, Valkonen JPT (2001). Organization of genes controlling disease resistance in the potato genome. Ann Rev Phytopathol.

[CR9] Haverkort AJ, Struik PC, Visser RGF, Jacobsen E (2009). Applied biotechnology to combat late blight in potato caused by *Phytophthora**infestans*. Potato Res.

[CR10] Huang S (2005) Discovery and characterization of the major late blight resistance complex in potato. PhD thesis Wageningen University, Wageningen

[CR11] Huang S, Vleeshouwers VG, Werij JS, Hutten RC, van Eck HJ, Visser RG, Jacobsen E (2004). The R3 resistance to *Phytophthora**infestans* in potato is conferred by two closely linked *R* genes with distinct specificities. Mol Plant Microbe Interact.

[CR12] Huang S, van der Vossen EA, Kuang H, Vleeshouwers VG, Zhang N, Borm TJ, van Eck HJ, Baker B, Jacobsen E, Visser RG (2005). Comparative genomics enabled the isolation of the *R3a* late blight resistance gene in potato. Plant J.

[CR13] Jo KR (2013) Unveiling and deploying durability of late blight resistance in potato from natural stacking to cisgenic stacking. PhD Thesis, Wageningen University, Wageningen, The Netherlands

[CR14] Khavkin EE, Sokolova EA, Beketova MP, Pankin AA, Kuznetsova MA, Kozlovskaya IN, Spiglazova SY, Statsyuk NV, Yashina IM. (2010) Potato resistance to late blight as related to the *R1* and *R3* genes introgressed from *Solanum**demissum*. In: Twelfth Euroblight Workshop Arras (France), 3–6 May 2010 PPO-Special Report 14:231–238

[CR15] Kim HJ, Lee HR, Kwang-Ryong J, Mortazavian SMM, Huigen DJ, Evenhuis B, Kessel G, Visser RGF, Jacobsen E, Vossen JH (2011). Broad spectrum late blight resistance in potato differential set plants MaR8 and MaR9 is conferred by multiple stacked *R* genes. Theor Appl Genet.

[CR16] Lees AK, Stewart JA, Lynott JS, Carnegie SF, Campbell H, Roberts AMI (2012). The effect of a dominant *Phytophthora**infestans* Genotype (*13_A2*) in Great Britain on host resistance to foliar late blight in commercial potato cultivars. Potato Res.

[CR17] Marczewski W, Strzelczyk-Żyta D, Hennig J, Witek K, Gebhardt C (2006). Potato chromosomes IX and XI carry genes for resistance to potato virus M. Theor Appl Genet.

[CR18] Orłowska E, Fiil A, Kirk HG, Llorente B, Cvitanich C (2012). Differential gene induction in resistant and susceptible potato cultivars at early stages of infection by *Phytophthora**infestans*. Plant Cell Rep.

[CR19] Orłowska E, Basile A, Kandzia I, Llorente B, Kirk HG, Cvitanich C (2012). Revealing the importance of meristems and roots for the development of hypersensitive responses and full foliar resistance to *Phytophthora**infestans* in the resistant potato cultivar Sarpo Mira. J Exp Bot.

[CR20] Pilet F, Pelle R, Ellisseche D, Andrivon D (2005). Efficacy of the R2 resistance gene as a component for the durable management of potato late blight in France. Plant Pathol.

[CR21] Rauscher G, Simko I, Mayton H, Bonierbale M, Smart CD, Grunwald NJ, Greenland A, Fry WE (2010). Quantitative resistance to late blight from *Solanum**berthaultii* cosegregates with *Rpi*-*ber*: insights in stability through isolates and environment. Theor Appl Genet.

[CR22] Rietman H, Bijsterbosch G, Cano LM, Lee HR, Vossen JH, Jacobsen E, Visser RG, Kamoun S, Vleeshouwers VG (2012). Qualitative and quantitative late blight resistance in the potato cultivar Sarpo Mira is determined by the perception of five distinct RXLR effectors. Mol Plant Microbe Interact.

[CR23] Śliwka J, Jakuczun H, Lebecka R, Marczewski W, Gebhardt C, Zimnoch-Guzowska E (2006). The novel, major locus *Rpi*-*phu1* for late blight resistance maps to potato chromosome IX and is not correlated with long vegetation period. Theor Appl Genet.

[CR24] Śliwka J, Świątek M, Tomczyńska I, Stefańczyk E, Chmielarz M, Zimnoch-Guzowska E (2013). Influence of genetic background and plant’s age on expression of potato late blight resistance gene *Rpi*-*phu1* during incompatible interaction with pathogen. Plant Pathol.

[CR25] Stewart HE, Bradshaw JE, Pande B (2003). The effect of the presence of *R*-genes for resistance to late blight (*Phytophthora**infestans*) of potato (*Solanum**tuberosum*) on the underlying level of field resistance. Plant Pathol.

[CR26] Świeżyński KM, Zimnoch-Guzowska E (2001). Breeding potato cultivars with tubers resistant to *Phytophthora**infestans*. Potato Res.

[CR27] Tan MYA, Hutten RCB, Celis C, Park T-H, Niks RE, Visser RGF, van Eck HJ (2008). The *Rpi*-*mcd1* locus from *Solanum**microdontum* involved in resistance to *Phytophthora**infestans*, causing a delay in infection, maps on potato chromosome 4 in a cluster of NBS-LRR genes. Mol Plant Microbe Interact.

[CR28] Tomczyńska I, Śliwka J (2010) Search for molecular markers linked to the late blight resistance of potato cultivar Sárpo Mira. In: Poster session presented at Pathology Section Meeting 2010 Potato Pests and Diseases: Old Enemies, New Threats, Carlow, 13–16 September

[CR29] Tresnik S (2007) State of the art of Integrated Crop Management & organic systems in Europe with particular reference to pest management Potato production. Pesticide Action Network (PAN) Europe: http://www.pan-europe.info/Resources/Reports/Potato_production_review.pdf

[CR30] Tschöpe B, Kleinhenz B, Keil S, Zellner M (2010) Öko-SIMPHYT (=Organic-SIMPHYT): A forecasting system for specific scheduling of copper fungicides against late blight. In: Twelfth Euroblight Workshop Arras (France), 3–6 May 2010 PPO-Special Report 14:153–158

[CR31] Twining S, Clarke J, Cook S, Ellis S, Gladders P, Ritchie F, Wynn S (2009) Research Report: Pesticide availability for potatoes following revision of Directive 91/414/EEC: Impact assessments and identification of research priorities: http://www.potato.org.uk

[CR32] Van Ooijen JW (2006) JoinMap ^®^ 4, Software for the calculation of the genetic linkage maps in experimental populations. Kyazma B.V., Wageningen, Netherlands

[CR33] Verzaux E (2010) Resistance and susceptibility to late blight in *Solanum*: gene mapping, cloning and stacking. PhD Thesis Wageningen University

[CR34] Verzaux E, van Arkel G, Vleeshouwers VGAA, van der Vossen EAG, Niks RE, Jacobsen E, Vossen J, Visser RGF (2012). High-resolution mapping of two broad-spectrum late blight resistance genes from two wild species of the *Solanum**circaeifolium* group. Potato Res.

[CR35] White S, Shaw D (2009). The usefulness of late-blight resistant Sárpo cultivars—a case study. Acta Hort (ISHS).

[CR36] White S, Shaw D (2010) Breeding for host resistance: The key to sustainable potato production. In: Twelfth Euroblight Workshop Arras (France), 3–6 May 2010 PPO-Special Report 14:125–132

[CR37] Zarzycka H (2001) Evaluation of resistance to *Phytophthora**infestans* in detached leaflet assay. Preparation of the inoculum. Plant Breeding and Acclimatization Institute, Radzików, Poland. IHAR Monografie i Rozprawy Naukowe 10a:75–77

[CR38] Zhu S, Li Y, Vossen JH, Visser RG, Jacobsen E (2012). Functional stacking of three resistance genes against *Phytophthora**infestans* in potato. Transgenic Res.

